# Vitamin C: From Bench to Bedside

**DOI:** 10.3390/nu13041102

**Published:** 2021-03-27

**Authors:** Anitra C. Carr, Jens Lykkesfeldt

**Affiliations:** 1 Nutrition in Medicine Research Group, Department of Pathology & Biomedical Science, University of Otago, Christchurch 8011, New Zealand; 2 Faculty of Health & Medical Sciences, University of Copenhagen, 1870 Frederiksberg, Denmark; jopl@sund.ku.dk

Vitamin C (ascorbic acid) is a normal liver metabolite in most animals, with humans being a notable exception due to random genetic mutations that have occurred during our evolution. As such, it has become a vitamin (vital to life), with requirements increasing significantly during various illnesses, particularly severe infections. Recent international clinical trials are highlighting the potential for intravenous vitamin C administration to improve clinical outcomes for patients, particularly those with severe respiratory illness and sepsis and some cancers. Furthermore, there has been an upsurge in new discoveries and new mechanistic insights, particularly around epigenetic regulation by vitamin C, that are providing rationales for future targeted clinical trials.

To facilitate the translation of leading-edge research into clinical practice, we held the second “Vitamin C Symposium” in 2019 with the theme of “Vitamin C for Cancer and Infection: From Bench to Bedside” [1]. The speakers were internationally renowned biomedical and clinical researchers and doctors with expertise in the fields of infection and cancer. The symposium was designed as an educational event targeted primarily at doctors and nurses and provided Continuing Professional Development through the Royal New Zealand College of General Practitioners (RNZCGP) and the College of Intensive Care Medicine of Australia and New Zealand (CICM). The content of the symposium comprised translational “bench to bedside” research, from laboratory-based experiments aimed at understanding the underlying mechanisms involved to clinical trials endeavoring to determine the efficacy of vitamin C in various infectious states and cancer types. This symposium was the inspiration for the Nutrients Special Issue “Vitamin C: from Bench to Bedside”.

In this Special Issue, we have collected 15 original research papers and comprehensive review articles spanning laboratory-based research, observational studies and intervention trials. These cover the following key research themes: pharmacokinetics, immune function, epidemiology, cancer research, surgical outcomes, and infection research, specifically pneumonia, sepsis, and COVID-19.

Pharmacokinetics: Lykkesfeldt and colleague wrote a comprehensive review highlighting the complex pharmacokinetics of oral and intravenous vitamin C [2], an issue that is often overlooked in the design and interpretation of clinical studies. Research carried out by Pullar and colleagues has indicated that due to the unique kinetics of vitamin C uptake into erythrocytes, these cells could potentially be used as an indicator of vitamin C status in nonfasting individuals [3], thereby overcoming the issue of plasma vitamin C fluctuations in response to recent dietary intake.

Immune function: Carr and colleagues investigated the role of vitamin C in neutrophil function, including a systematic review of randomized controlled trials carried out in different population groups and patient cohorts [4]. The neutrophil functions assessed included chemotaxis, phagocytosis, oxidative burst activity, enzyme activity, and apoptosis. Additional laboratory-based research showed that enhanced uptake of ascorbate by human neutrophils can affect key functions, including chemotaxis and neutrophil extracellular trap (NET) formation [5].

Epidemiology: Dr Myint and colleagues interrogated the large European Prospective Investigation into Cancer-Norfolk (EPIC-Norfolk) database to determine demographic and lifestyle risk factors for vitamin C deficiency and associations with functional health [6]. They found that vitamin C deficiency was associated with older age, being male, lower physical activity, smoking, being more socially deprived and a lower educational attainment. Those in the lowest quartile of vitamin C were also more likely to score in the lowest decile of physical function, bodily pain, general health, and vitality.

Cancer research: Dr Wang and colleagues have identified key molecular targets of vitamin C activity in triple-negative breast cancer metastasis in cell culture and a xenograft model [7], indicating that vitamin C can potentially inhibit metastasis by modulating the expression of SYNPO2 and YAP1. Observational research by Carr and colleagues has indicated that patients undergoing chemotherapy in association with stem cell transplantation exhibit depleted vitamin C status in association with febrile neutropenia [8]. Further observational research by Dachs and colleagues indicated that low vitamin C status in patients with cancer is associated with both patient and tumor characteristics [9].

Surgical outcomes: A systematic review on the effects of vitamin C administration on outcomes of Percutaneous Coronary Intervention (PCI) has indicated that intravenous infusion of vitamin C before PCI may serve as an effective method for cardioprotection against reperfusion injury [10]. A meta-analysis on the effects of perioperative vitamin C administration on postoperative analgesia consumption indicated a lower pain score and a lower morphine consumption in those receiving intravenous vitamin C, but not oral vitamin C [11].

Pneumonia and sepsis: Observational research by Carr and colleagues has indicated that hospitalized patients with community-acquired pneumonia exhibit both depleted vitamin C status and elevated markers of oxidative stress [12]. Pneumonia is a major driver for the development of sepsis. Harrison and colleagues reported enhanced synthesis of vitamin C in mice following a septic insult [13], but despite this, there was still a decrease in vitamin C levels in the brain, as well as upregulation of key inflammatory cytokines (IL-6, IL-1β, TNFα) and chemokines (CXCL1, KC/Gro). Kim et al. retrospectively identified specific subphenotypes in patients with septic shock that may respond differently to treatment with vitamin C, hydrocortisone and thiamine combination therapy [14]. They indicated that clinical outcomes might be better for patients with the hyperinflammatory subphenotype.

Sepsis and COVID-19: Dr Fowler and colleagues wrote a comprehensive review on the emerging role of vitamin C as a treatment for sepsis [15], which covers the pleiotropic functions of vitamin C in sepsis and acute respiratory distress syndrome (ARDS) and includes a discussion of their recent CITRIS-ALI trial which showed decreased mortality in sepsis-induced acute lung injury (ALI) following administration of intravenous vitamin C. As highlighted in the accompanying Editorial [16], this review is particular pertinent in light of the global SARS-CoV-2 pandemic as pneumonia and sepsis are major complications of severe Coronavirus Disease-2019 (COVID-19). Of note, preliminary observational and interventional studies are indicating a potential role for vitamin C in the prevention and treatment of COVID-19 [16].

Collectively, this Special Issue displays the diverse areas of health and disease in which vitamin C plays important roles and the continuing efforts of researchers to unravel the underlying mechanisms of its biological functions. Although its role in scurvy has been recognized for close to a century, it appears that we still have much to learn about this small carbohydrate molecule. Well-designed observational and interventional studies that consider the baseline status and unique pharmacokinetics of vitamin C are needed moving forward to help address the current gaps in our knowledge. Underpinning mechanistic research will also further our ability to inform good clinical practice. We look forward to future discoveries of the roles of vitamin C in human health and disease.

## Data Availability

Not applicable.
